# Chemical Constituents of a Marine-Derived Endophytic Fungus *Penicillium commune* G2M

**DOI:** 10.3390/molecules15053270

**Published:** 2010-05-04

**Authors:** Hui-Jiao Yan, Shu-Shan Gao, Chun-Shun Li, Xiao-Ming Li, Bin-Gui Wang

**Affiliations:** 1 Key Laboratory of Experimental Marine Biology, Institute of Oceanology, Chinese Academy of Sciences, Qingdao 266071, China; E-Mails: yanhuijiao@yahoo.com.cn (H.-J.Y.); xisea01@126.com (S.-S.G.); lichunshun@ms.qdio.ac.cn (C.-S.L.); lixmqd@yahoo.com.cn (X.-M.L.); 2 Graduate School of the Chinese Academy of Sciences, Beijing 100049, China

**Keywords:** chemical constituents, endophytic fungus, *Hibiscus tiliaceus*, *Penicillium commune*

## Abstract

Cultivation of the endophytic fungus *Penicillium commune*, which was isolated from the semi-mangrove plant *Hibiscus tiliaceus*, afforded one new compound 1-*O*-(2,4-dihydroxy-6-methylbenzoyl)-glycerol (**1**) along with thirteen known products, including 1-*O*-acetylglycerol (**2**), *N*-acetyltryptophan (**3**), 3-indolylacetic acid methyl ester (**4**), 1-(2,4-dihydroxy-3,5-dimethylphenyl)ethanone (**5**), 2-(2,5-dihydroxyphenyl)acetic acid (**6**), (4*R*,5*S*)-5-hydroxyhexan-4-olide (**7**), thymidine (**8**), uracil (**9**), thymine (**10**), ergosterol (**11**), *β*-sitosterol (**12**), *β*-daucosterol (**13**), and ergosta-7,22-dien-3*β*,5α,6*β*-triol (**14**). The structures of these compounds were established by detailed NMR spectroscopic analysis, as well as by comparison with literature data or with authentic samples.

## 1. Introduction 

Mangrove and semi-mangrove plants are mostly distributed in the tropical and subtropical coastal regions of the world. Because of their special ecosystems that straddle the land and the sea, mangrove plants are found to be a rich source of mutualistic fungal endophyte microorganism species [1,2]. Our previous investigations on the mangrove-derived fungi *Eurotium rubrum* have led to the isolation of a variety of structurally unique metabolites with different biological activities [3,4,5]. During our ongoing search for structurally interesting metabolites from mangrove-derived fungi, a strain of *Penicillium commune* G2M, which was isolated from the semi-mangrove plant *Hibiscus tiliaceus* collected from Hainan Island of China, was investigated. As a result, one new and 13 known metabolites with diverse molecular structures were isolated and identified from the rice fermentation culture of the strain. This paper describes the isolation and structure elucidation of these compounds.

## 2. Results and Discussion 

Compound **1** was obtained as pale yellow oil. The IR absorptions at 3,440 and 3,274, 1,716, and 1,623 cm^–1^ indicated the presence of hydroxyl, carbonyl, and aromatic groups in the molecule, respectively. The molecular formula was determined to be C_11_H_14_O_6_ on the basis of HRESIMS (*m/z* 265.0689 [M + Na]^+^, calcd. for C_11_H_14_O_6_Na^+^, 265.0688), which was in agreement with the ^1^H- and ^13^C-NMR spectral data (Table 1). 

**Figure 1:**
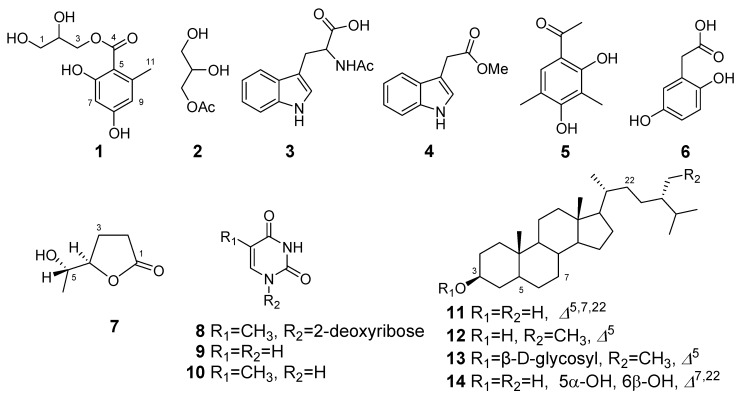
Chemical structures of compounds **1**-**14**.

The ^13^C-NMR and DEPT spectra displayed the presence of one methyl, two oxymethylenes, one oxymethine, one carbonyl, and six aromatic carbon groups in the molecules. The characteristic ^1^H-NMR resonances clearly indicated the presence of two aromatic *meta*-protons at *δ*_H_ 6.15 (1H, d, *J* = 2.5 Hz, H-7) and 6.21 (1H, d, *J* = 2.5 Hz, H-9). The HMBC correlations from the methyl H_3_-11 (*δ*_H_ 2.50) to C-5 (*δ*_C_ 106.0), C-9 (*δ*_C_ 112.5), and C-10 (*δ*_C_ 144.7) located the methyl group at C-10. In addition, the observed HMBC cross peaks from H-7 to C-5, C-6 (*δ*_C_ 166.1), C-8 (*δ*_C_ 163.8), and C-9, and from H-9 to C-5, C-7 (*δ*_C_ 101.8), and C-11 (*δ*_C_ 24.4) unambiguously suggested the substitution mode of the benzene ring. On the other hand, the ^1^H-^1^H COSY spectrum indicated that all aliphatic oxymethylene and oxymethine protons were part of a contiguous spin system comprising H_2_-1 (*δ*_H_ 3.63), H-2 (*δ*_H_ 3.97), and H_2_-3 (*δ*_H_ 4.31 and 4.42). Finally, the observed HMBC correlations from H_2_-3 to the carbonyl carbon C-4 (*δ*_C_ 172.8) established the connection of glyceryl and 2,4-dihydroxy-6-methylbenzoyl units via ester linkage. Thus, the structure of **1** was determined as 1-*O*-(2,4-dihydroxy-6-methylbenzoyl)-glycerol, as shown in Figure 1.

Besides the new compound **1**, another 13 known metabolites, including 1-*O*-acetylglycerol (**2**) [6], *N*-acetyltryptophan (**3**) (determined by comparison with spectral data of *N*-acetyltryptophan methyl ester) [7], 3-indolylacetic acid methyl ester (**4**) [8], 1-(2,4-dihydroxy-3,5-dimethylphenyl)ethanone (**5**, known as clavatol) [9], 2-(2,5-dihydroxyphenyl)acetic acid (**6**) [10], (4*R*,5*S*)-5-hydroxyhexan-4-olide (**7**) [11], thymidine (**8**) [12], uracil (**9**) [12], thymine (**10**) [12], ergosterol (**11**) [13], *β*-sitosterol (**12**), *β*-daucosterol (**13**), and ergosta-7,22-dien-3*β*,5α,6*β*-triol (**14**) [14], were also isolated and identified from the rice fermentation culture of *Penicillium commune*. Their structures are shown in Figure 1.

## 3. Conclusions

In summary, we have identified a new glycerol derivative 1-*O*-(2,4-dihydroxy-6-methylbenzoyl)-glycerol (**1**), together with thirteen known products **2**–**14** of diverse molecular types from the solid cultures of the endophytic fungal strain *P. commune* that was isolated from the semi-mangrove plant *Hibiscus tiliaceus*. The fungal species *P. commune* are widely spread in nature, being isolated usually from foods and plant rhizospheres. The earlier studies showed that strains of *P. commune* mainly produce indole alkaloids such as cyclopiazonic acid [15,16], fumigaclavines A and B [17], penitrem A and roquefortine [18]. Up to the present, there are no reports of the chemical constituents of mangrove-derived *P. commune* and this is the first report of the isolation of glycerol derivative such as compound **1** from the fungal genus *Penicillium*. Our chemical study of the mangrove-derived endophyte *P. commune* enriches the chemical diversity of this fungal species.

## 4. Experimental

### 4.1. General

Optical rotations were measured on a AA-55 polarimeter (Optical Activity Ltd). IR spectra were performed on a JASCO FT/IR-4100 Fourier Transform infrared spectrometer. UV spectra were measured on a Lengguang Gold spectrumlab 54. NMR spectra were recorded on a Bruker Avance 500 MHz spectrometer (500 MHz for ^1^H and 125 MHz for ^13^C) and chemical shifts were recorded as *δ* values. Mass spectra were performed on a VG Autospec 3000 mass spectrometer. Silica gel (200–300 mesh, Qingdao Haiyang Chemical Co., Qingdao, China), reversed-phase silica gel C18 (40–75 mm, Fuji Silysia Chemical Ltd.) and Sephadex LH-20 (18–110 mm, Merck, Darmstadt, Germany) were used for open column chromatography (CC).

### 4.2. Fungal material

*Penicillium commune* G2M was isolated from a sample of the mangrove plant *Hibiscus tiliaceus* Linn. that was collected from Hainan Island, China, in August, 2004, by using the procedures described in our previous report [3,4,5]. Fungal identification was carried out using a molecular biological protocol of DNA amplification and sequencing of the ITS region as described previously [3,4,5]. The sequence data obtained from the fungal strain has been submitted to and deposited at GenBank under accession no. HM064435. A BLAST search result indicated that the sequence was the most similar (98%) to the sequence of *Penicillium commune* (compared to GQ 340555.1, GI 260986214). The strain is preserved at 4 °C at the Key Laboratory of Experimental Marine Biology, Institute of Oceanology, Chinese Academy of Sciences. The fungal strain was statically fermented at room temperature on sterilized solid medium containing 100 g rice, 0.6 g peptone, and 100 mL sea water, in 1 L Fernbach flasks (×100) for 42 days.

### 4.3. Extraction and isolation

The fermented rice substrate was extracted repeatedly with EtOAc, and the organic solvent was evaporated to dryness under vacuum to afford 20 g of crude extract. The extract was subjected to CC over silica gel eluted with petroleum ether (PE)–EtOAc (from 1:0 to 1:1) and CHCl_3_–MeOH (from 20:1 to 0:1) to yield seven fractions (Frs. 1–7). Fr. 2 was further purified by Sephadex LH-20 (CHCl_3_–MeOH 1:1) to afford **5** (15.8 mg). Fr. 3 was fractionated by CC on silica gel eluted with PE–EtOAc (from 100:1 to 10:1) to obtain **11** (20.0 mg) and **12** (8.9 mg). Fr. 4 was subjected to CC on silica gel eluted with PE–EtOAc (from 50:1 to 10:1), and RP-18 (MeOH) to yield **7** (5.4 mg). Fr. 5 was subjected to Sephadex LH-20 (CHCl_3_–MeOH 1:1) and RP-18 eluted with MeOH–H_2_O (0–100) to give **3** (4.2 mg), **8** (32.6 mg), **13** (19.3 mg), and **14** (6.1 mg). Fr. 6 was further purified by Sephadex LH-20 (CHCl_3_–MeOH 1:1), Sephadex LH-20 (MeOH), and RP-18 with MeOH–H_2_O (0–100) as an eluent to afford **1** (4.1 mg), **2** (19.3 mg), **6** (31.1 mg), **9** (6.1 mg), and **10** (7.3 mg). Fr. 7 was further purified by Sephadex LH-20 (CHCl_3_–MeOH 1:1) to yield **4** (3.4 mg).

*1-O-(2,4-Dihydroxy-6-methylbenzoyl)-glycerol* (**1**): pale yellow oil; [α]^25^_D_: + 15° (c 0.4, MeOH); IR (KBr) cm^–1^: 3,440, 3,274, 3,070, 2,939, 1,716, 1,623, 1,450, 1,381, 1,323, 1,261, 1,192, 1,115; UV λ_max_ (MeOH) nm (log *ε*): 215 (4.63), 264 (4.40); ^1^H-NMR and ^13^C-NMR: see Table 1; ESI-MS: 265 [M + Na]^+^, 507 [2M + Na]^+^; HR-ESI-MS: *m/z* 265.0689 [M + Na]^+^, calcd. for C_11_H_14_O_6_Na^+^, 265.0688.

## Figures and Tables

**Table 1:** 1D and 2D NMR data of **1** (in CD_3_OD, *δ* in ppm, *J* in Hz).

	^13^C (DEPT)	^1^H (mult, *J* in Hz)	^1^H-^1^H COSY	HMBC
1	64.4 (t)	3.63 (m)	H-2	C-2, 3
2	71.1 (d)	3.97 (m)	H-1, H_2_-3	C-1, 3
3	67.1 (t)	3a: 4.31 (dd, 11.5, 6.0)	H-2	C-1, 2, 4
		3b: 4.42 (dd, 11.5, 4.5)	H-2	C-1, 4
4	172.8 (s)	-	-	-
5	106.0 (s)	-	-	-
6	166.1 (s)	-	-	-
7	101.8 (d)	6.15 (d, 2.5)	H-9	C-5, 6, 8, 9
8	163.8 (s)	-	-	-
9	112.5 (d)	6.21 (d, 2.5)	H-7	C-5, 7, 8, 11
10	144.7 (s)	-	-	-
11	24.4 (q)	2.50 (s)	-	C-5, 9, 10
